# Letter to the Editor: comment on “Comorbidity of cardiac metabolism and increased risk of postoperative delirium – perhaps mediated by multiple factors”

**DOI:** 10.1097/JS9.0000000000003014

**Published:** 2025-07-08

**Authors:** Wenyu Guo, Xin Xin, Jiaxi Li

**Affiliations:** a Shandong Wendeng Osteopathic Hospital, Institute of Orthopedics and Traumatology, Shandong, Wendeng, China; b Shanghai University of Traditional Chinese Medicine, Shanghai, China; c Shandong Wendeng Osteopathic Hospital, Shandong Institute of Orthopedics and Traumatology, Shandong, Wendeng, China

*Dear Editor,*

We read with great interest the recent article by Wang Kun *et al* titled “Association of Cardiometabolic Multimorbidity with Postoperative Delirium and Three-Year Mortality in Patients Undergoing Knee/Hip Replacement: A Prospective Cohort Study”^[1]^. The authors conducted a range of analyses using data from the Perioperative Neurocognitive Disorders and Lifestyle Biomarkers in Elderly Patients (PNDABLE) database, which revealed a preliminary association between cardiometabolic comorbidities and postoperative delirium. Additionally, through mediation analysis, the study explored the mediating role of cerebrovascular fluid biomarkers in this association, offering potential insights for clinical treatment and prevention strategies. While the authors acknowledge the limitations of their research, we believe that several aspects merit further discussion, as they may impact the interpretation of the current findings and guide future research directions:

Firstly, due to insufficient consideration of potential confounding factors, the study would benefit from more rigorous inclusion and exclusion criteria. The participants in this study underwent knee or hip replacement surgery, and the diversity of indications for these procedures—such as osteoarthritis, femoral head necrosis, rheumatoid arthritis, and congenital underdevelopment—determines the differences in participants’ underlying health conditions. For instance, preoperative inflammation levels have been identified as a risk factor for postoperative delirium^[2]^, while chronic pain-related fatigue may lead to fluctuations in cerebrospinal fluid biomarkers^[3]^. These factors highlight the importance of thoroughly examining the reasons for joint replacement surgery among enrolled individuals. Furthermore, relevant studies have suggested a potential association between the use of tourniquets during surgery and postoperative delirium^[4]^. Therefore, to obtain more rigorous conclusions, it is crucial to consider the diversity of surgical indications, conduct strict preoperative assessments of pain and fatigue, and implement unified intraoperative management methods.

Secondly, there are notable deficiencies in the research methodological design. First, the study’s type appears unclear, as the distinction between a prospective cohort study and a retrospective clinical study is significant. Additionally, the research results indicated that patients in the postoperative delirium group and the non-postoperative delirium group had significantly different ages. Specifically, the postoperative delirium group included individuals with a higher average age, and age was found to have a significant correlation with cardiometabolic comorbidities^[5]^. This raises the question of whether the associations identified in this study might largely be driven by aging, given that age also influences levels of various cerebrovascular fluid biomarkers^[6]^. The exclusion of age as a variable in the logistic regression analysis further increases the risk of bias, highlighting the need for age stratification and subgroup analysis. Moreover, as a retrospective study, there was a notable disparity in sample size between the two groups. To address this, employing propensity score matching to screen and match patients with similar baseline characteristics, such as age, could enhance the study’s internal validity and more closely approximate the outcomes of a randomized controlled trial^[7]^.

Finally, the insufficient sample size undermines the credibility and generalizability of the conclusions. The actual number of included patients fell short of the anticipated sample size, leading to inadequate statistical power, particularly in the post hoc and survival analysis sections. Moreover, all patients were sourced from a single database, which restricts the study’s external validity. Expanding the database sources to include more patients or validating the findings with external datasets could yield more robust and convincing conclusions.

In summary, caution must be exercised when interpreting the study’s results, and more statistically robust analysis methods should be employed to evaluate the association between cardiometabolic comorbidities and postoperative delirium following joint replacement surgery, especially when it comes to the mediating effects of cerebrospinal fluid biomarkers based on this association, special attention should be given to assessing the mediating role. Otherwise, it may result in misleading conclusions that are difficult to generalize to clinical practice.

## Data Availability

Data availability is not applicable to this article as no new data were created or analyzed in this study.
